# Extra-articular Tenodesis for ACL Reconstruction: Who Needs it and is there a Superior Technique?

**DOI:** 10.1007/s12178-026-10028-9

**Published:** 2026-04-02

**Authors:** Enejd Veizi, Lucy Oliver-Welsh, Christos Koutserimpas, Emrah Imat, Alan Getgood

**Affiliations:** 1 Department of Surgery, Aspetar Orthopaedic and Sports Medicine Hospital, Doha, Qatar; 2 Department of Orthopedics and Traumatology, Ankara Yıldırım Beyazıt University, Ankara, Turkey; 3 Brisbane Orthopaedic & Sports Medicine Centre, Brisbane Private Hospital, Spring Hill, Brisbane, Queensland Australia; 4 Orthopaedics Surgery and Sports Medicine Department, FIFA Medical Center of Excellence, Croix-Rousse Hospital, Lyon University Hospital, Hospices Civils de Lyon, Lyon, France; 5 School of Rehabilitation Health Sciences, University of Patras, Patras, Greece; 6 Department of Orthopedics and Traumatology, Etlik City Hospital, Ankara, Turkey; 7 FIFA Medical Centre of Excellence, Fortius Clinic, London, UK

**Keywords:** Lateral extra-articular tenodesis, Anterior cruciate ligament, Anterolateral complex, Ligament reconstruction, Arthroscopy, Knee

## Abstract

**Purpose of review:**

To provide a comprehensive overview of the indications for lateral extra-articular tenodesis in addition to anterior cruciate ligament reconstruction (ACLR), followed by a discussion on the existing techniques with their perceived advantages and disadvantages.

**Recent findings:**

Recent evidence has shifted lateral extra-articular tenodesis (LET) from a routine “add-on” to a selective adjunct for patients at higher risk of residual rotatory instability and ACL graft failure after isolated ACLR. Level I clinical evidence suggests that adding an iliotibial (ITB) – based LET can meaningfully reduce graft rupture rates while improving control of pivot shift – type instability. The 2025 International Consensus on lateral extra-articular procedures (LEAPs) supports LET/anterolateral augmentation particularly for revision ACLR, high-grade pivot shift, generalized laxity or hyperextension/recurvatum, and young athletes returning to pivoting/contact sports. The consensus also mentions that modern LEAPs have low complication rates, do not typically require altered rehabilitation, and have not shown a consistent signal for increased lateral compartment osteoarthritis when contemporary technique principles are followed. Finally, despite the variety of described constructs (modified Lemaire variants, other ITB-based tenodeses, and anatomic ALL reconstruction), current literature does not demonstrate a single universally superior technique. Instead, success appears to be linked to correct patient selection, adherence to key technical principles (appropriate graft routing & low-tension fixation), and age or skeletal – maturity – appropriate modifications.

**Summary:**

When applied selectively in high-risk patients, adding a lateral extra-articular tenodesis to ACL reconstruction can improve rotational control and reduce re-injury, but current evidence does not support a single universally superior LET construct. Optimal results depend on appropriate indications, careful technique, and tailoring the approach to patient factors such as age, sport demands, laxity profile, and skeletal maturity.

## Introduction

The anterolateral complex (ALC) structures, which is comprised of the anterolateral ligament (ALL), the anterolateral capsule, the Kaplan fibers and the iliotibial band, have all been shown to be responsible for regulating tibial internal rotation during knee motion [1, 2]. Anterior cruciate ligament (ACL) injury is seldomly isolated, often presenting with concomitant ALC injuries [3, 4]. Residual rotatory instability of the knee joint after an ACL rupture is a complex phenomenon involving intra-articular and extra-articular structures of the joint [5]. Surgical ACL reconstruction (ACLR) is considered the gold standard procedure in active patients with a desire to return to sporting activity and there is mounting evidence in the literature that an isolated ACLR procedure might be insufficient in mitigating the additional sustained injuries of the joint, leading to persistent instability and potential graft re-rupture [3, 6, 7].

Lateral extra-articular tenodesis (LET) in addition to ACLR, while not a new concept, has been the focus of extensive research in the last decade [5]. Seen as a reinforcement to residual knee rotational laxity after an ACL injury, extra-articular procedures provide increased stability and act as a biomechanically load-sharing medium until graft healing is completed [8, 9]. The results of the STABILITY trial, which focused on young patients at high risk of failure (aged 14–25), showed that graft rupture occurred in 4% of patients when an LET was added to the ACLR, compared to 11% when it was not [10]. Age alone has been shown by multiple authors to be the leading risk factor for graft re-rupture after ACLR [11–13] but the indications for LET have expanded to include joint hyperextension, skeletal immaturity, overall activity status and chronic ACL deficiency [3, 14].

There are many techniques described in the literature focused on augmenting or reconstructing the ALC and at least as many fixation methods to stabilize the graft or iliotibial flaps to the femur [8]. The purpose of this narrative review is to provide a comprehensive overview of the indications for lateral extra-articular tenodesis in addition to anterior cruciate ligament reconstruction, followed by a discussion on the existing techniques with their perceived advantages and disadvantages.

### Historical Overview

Early concepts underlying lateral extra-articular augmentation predate modern arthroscopic ACL reconstruction and arose from efforts to explain rotatory knee instability. Late-19th-century observations such as Segond’s description of a characteristic lateral avulsion injury helped frame the anterolateral side of the knee as clinically relevant in rotational trauma patterns [15]. In the modern ligament era, Slocum and Larson formalized the clinical problem of rotatory instability in ACL-deficient knees [16], and subsequent work by Galway and MacIntosh popularized the pivot-shift as a practical sign of symptomatic anterolateral rotatory instability [17]. These milestones created a clear therapeutic target; restoring rotational control when isolated intra-articular approaches were either unavailable or insufficient.

Within this historical context, French surgeon Marcel Lemaire is widely credited with the first detailed description of an isolated lateral extra-articular tenodesis (LET) using the iliotibial band to improve rotatory stability in the 1960s [18]. Multiple refinements and modifications followed, notably MacIntosh and Darby’s lateral extra-articular concepts [19], with a later integrated into combined intra-/extra-articular “over-the-top” reconstructions, and Ellison’s distally based iliotibial-band transfer (late 1970s) [20]. Despite broad adoption in the 1970–1980 s, enthusiasm declined after the 1989 AOSSM consensus, which viewed routine extra-articular procedures as offering limited added value and potentially higher morbidity compared with evolving intra-articular reconstructions [6, 21]. Interest resurged in the 2010s after renewed anatomic focus on the anterolateral region, including the ALL description by Claes et al. [22], and clinical data suggesting anterolateral complex injury is common in acute ACL rupture [23]. This culminated in the international consensus work positioning LET as a selective adjunct, particularly for revision cases and patients with high-grade pivot shift, generalized laxity/hyperextension, and young athletes returning to pivoting contact sports [24].

### Anatomical Summary

First described by Segond in 1879 as a fibrous band which seemed to tension with extensive internal rotation of the tibia and deemed responsible for what was later called the Segond fracture, the anterolateral ligament was long overlooked by the surgical community [1]. It’s association with ACL ruptures was brought again to attention after Claes et al. [22] showed that this structure, which he named as the anterolateral ligament (ALL), could be identified in 97% of all specimens. This claim has since been challenged [2, 25].

The anterolateral complex of the knee has been described as a three-layered structure extending from the distal lateral femur to the proximal lateral tibia and augmented by the presence of the iliotibial band (ITB) [26]. Herbst et al. [2, 27] in their detailed studies on the ALC describe three major layers with Layer 1 being the deep fascia, dominated anterolaterally by the iliotibial band and posterolaterally by the biceps femoris fascia. The ITB is divided into superficial and deep ITB, within Layer 1. Layer 2 comprises the retinacula and quadriceps aponeurotic expansions, including lateral patellofemoral ligamentous structures, and is incomplete posteriorly while blending with Layer 1 more anteriorly. Layer 3 is the true joint capsule, which around the lateral collateral ligament (LCL) region is organized into superficial and deep laminae (with loose tissue and vessels between them) that fuse anterior to the LCL.

The superficial and deep ITB are reinforced by Kaplan fiber attachments to the distal femur, and further supported by a distinct capsulo-osseous layer that is continuous with surrounding posterolateral fascial tissues such as lateral gastrocnemius and the biceps femoris and merge distally with the other ITB components toward the tibial insertions. Distally, these ITB-related elements become confluent, spanning an insertional footprint from the Gerdy tubercle anteriorly toward a more posterior lateral tibial attachment.

Beneath the ITB layers, the remaining anterolateral capsule (Layer 3) is a laminar sheet; the superficial capsular layer envelops the LCL, the deep layer runs deep and medial to it, and anterior to the LCL these layers unite—an anatomic relationship that can produce a mid-third capsular thickening in some knees and reported as present in 35% of specimens in a fresh-frozen series [1, 2]. This capsular thickening situated between the ITB and LCL, sometimes reported as the ALL, has been found to have attachments to the lateral meniscus, both as a proximal meniscofemoral attachment and as a distal meniscotibial attachment [28]. The link has been stipulated to have load-sharing properties between the lateral meniscus and the mentioned capsular thickening [22, 29].

Rotational control is best understood as the behavior of an integrated ITB–capsule complex, not a solitary ‘ALL’. Not all studies have identified a discrete ligament uniformly matching classic ALL descriptions and most of them argue that many similar reports likely correspond to either the capsulo-osseous ITB layer or the mid-third capsular thickening [30, 31].

Despite the debates, what is known is that the ALC plays a pivotal role in stabilizing and restricting excessive internal rotation of the tibia over the femur. This has led to studies focused on the biomechanical properties of this region of the knee, especially since the incidence of ACL injuries continues to increase and residual rotatory is seen as one of the main causes of graft re-rupture [1, 2].

### Biomechanics

Classic biomechanical studies have established the ACL as the primary restraint to anterior tibial translation and a major restraint to tibial internal rotation. Residual anterolateral rotatory laxity after ACL injury has driven renewed attention to the lateral soft tissues of the ALC. Clinically, this rotatory laxity is most commonly observed as a positive residual pivot shift, which in reality, is an anterolateral subluxation of the lateral tibiofemoral compartment and subsequent reduction [32, 33].

The ALL was historically viewed as a discrete structure with a clear role in controlling internal rotation and, to a lesser extent, anterior translation [22]. Contemporary anatomic and experimental studies instead support that the “ALL” often behaves as a capsular thickening with variable femoral attachment descriptions, which likely contributes to heterogeneity in reported function. Cadaveric studies have shown that the ALL is taut in internal rotation at around 30° of knee flexion, broadly paralleling the flexion-dependent behavior of the ACL [32, 34]. Its contribution to resisting anterior drawer, on the other hand, is consistently far smaller than the ACL. Reported length-change behavior is mixed and differences in landmark definitions (anterior vs. posterior to the lateral epicondyle) plausibly shift the ligament’s functional envelope across specimens [24, 32, 34].

Methodological factors help explain the conflicting sectioning data in the literature. Several experiments report minimal changes in global knee laxity after isolated ALL sectioning, especially when other major lateral stabilizers remain intact. Robotic and coupled-loading models in ACL-deficient knees more consistently show that compromising anterolateral tissues increases internal rotation and/or pivot-shift–type laxity [4, 35, 36]. Collectively, these findings support the ALL as a secondary stabilizer whose measurable effect becomes most apparent when the ACL is deficient and when loading reproduces the coupled translations and rotations of clinical laxity tests [37].

Rotatory stability is shared across multiple structures and concomitant pathology such as meniscal injury or capsular disruption can amplify abnormal kinematics and may contribute to residual pivot shift even after otherwise anatomic ACL reconstruction [16, 35]. Accordingly, the modern emphasis has shifted more towards the anterolateral complex which includes the ALL and anterolateral capsule together with the iliotibial band (ITB) and its deep/capsulo-osseous components [4, 35].

High-fidelity robotic cadaveric studies suggest that the ITB complex is often the dominant extra-articular restraint to internal tibial rotation, particularly at higher flexion angles, and can contribute substantially to controlling pivot-shift kinematics in ACL-deficient knees [9, 38]. According to the authors, the ALL/anterolateral capsule provides a smaller but non-negligible secondary contribution. These biomechanical data provide the logical rationale for ALL reconstruction or lateral extra-articular tenodesis.

### Indications vs. Complications

Contemporary use of lateral extra-articular tenodesis has shifted from a “routine add-on” to a targeted addition for patients at high risk of residual rotatory laxity or graft failure after isolated ACLR. Importantly, the indications most commonly cited today reflect the conclusions of the International Consensus meeting of 2025 [3, 14], which emphasized that LET should be considered primarily in higher-risk situations rather than universally. In practical terms, those consensus-driven indications cluster around;

1. high-grade rotational laxity on examination (typically grade 2–3 pivot shift)
2.  revision ACL reconstruction
3. young patients returning to pivoting/contact sports, especially when additional risk factors for failure are present.

Additional clinical scenarios frequently considered alongside these core indications include:

**Table:** 

• generalized ligamentous laxity	• meniscal deficiency or complex meniscal pathology
• genu recurvatum	
• hyperextension	• increased posterior tibial slope
• chronic symptomatic ACL deficiency	• prior contralateral ACL rupture.

Overall, the modern rationale is risk-mitigation; LET is used to improve rotatory control and reduce the burden on the intra-articular graft in patients whose baseline risk profile makes isolated ACLR more likely to fail or leave clinically meaningful instability.

At the same time, the decision is rarely binary, because “borderline” indications often accumulate. The addition of an LET is recommended when multiple relative risk factors coexist even when no single feature alone mandates augmentation e.g. pivoting sport exposure, meniscal deficiency, generalized laxity, increased tibial slope, prior contralateral injury, or clinical signs suggesting a strong rotational component. Conversely, there remains variability in whether LET should be added for isolated factors such as small graft diameter, female sex, imaging findings of anterolateral injury (e.g., Segond fracture or lateral femoral notch sign), or concomitant meniscal procedures; areas where evidence is evolving and practice patterns differ. As a result, many authors emphasize individualized thresholds rather than a single universal algorithm.

With respect to complications, modern LET performed alongside ACLR is generally described as safe with a low overall complication rate and without a clear signal of increased lateral-compartment osteoarthritis when contemporary technique and tensioning principles are followed. The main practical risks are usually local and related to hardware irritation [39, 40]. Getgood et al. [10] reported that the ACLR combined with an LET procedure had a slightly higher rate of hardware removal rates but the overall incidence of minor medical and surgical events remained low. This has been later supplemented by studies showing greater pain at the 3-months post operative time point, but with the difference not being clinically significant at later follow-up appointments and with similar range of motion angles between the groups [41]. Tunnel convergence has been mentioned as a potential complication when a screw is used to fix the ITB graft [42] but this was not confirmed in subsequent meta-analyses [43]. Overall additional complications such as hematoma, stiffness, arthrofibrosis, infection and capsular injury have also been reported at similar frequencies with isolated ACLR procedures [14, 40, 43].

Many of these adverse events can be minimized by avoiding increased constraint of physiologic motion of the ITB graft. While there is no clear consensus on the fixation angle [44], the International Consensus supports a fixation at a flexion angle between 0–60^o^, in neutral rotation and with a low tension [14]. The ITB graft should be passed deep to the lateral collateral ligament and the fixation point was recommended to be posterior and proximal to the femoral epicondyle, allowing the LET to be tight in extension and slack in flexion. On the topic of fixation method for the ITB graft, biomechanical studies have shown that staple fixation could result in early failure when compared to both interference screw and anchor fixations [45]. Clinically this has not been proven to be significant with all fixation methods showing similar overall performance [14].

Overloading of the lateral compartment has been an additional concern after an LET procedure [46]. Contrary to expectations, Gkekas et al. [7] in their recent systematic review showed that that LET significantly reduces moderate-to-severe OA risk, particularly in the lateral compartment and among knees with a history of meniscectomy. They actually suggested that usage of LET during primary ACLR with compromised meniscal integrity could help mitigate OA progression. Additionally, Ackermann et al. showed that static tibio-femoral rotation remained unchanged after ACLR, regardless of LET usage, suggesting LET does not modify rotational alignment in a static unloaded joint [47]. Focusing on the patellofemoral (PF) joint, Nakanishi et al. [48] found no difference in PF cartilage health between knees 2 years after primary ACLR with hamstring tendon autograft with or without LET. On a different note, Firth et al. [49] in 2024, using quantitative MRI views, showed early biochemical changes in the articular cartilage of the anterolateral compartment after an ACLR and LET procedure. While the clinical impact of these changes is not completely clear, we do not see clinically concerning changes in either the lateral compartment or the PF compartment with the addition of LET.

Finally, current expert opinion also supports that adding LET does not usually require a different rehabilitation progression or delayed return-to-sport timeline compared with standard ACLR, provided the intra-articular reconstruction and meniscal/cartilage procedures allow routine rehab [14].

### Current techniques

#### Modified Lemaire Procedure

The addition of the modern “modified Lemaire” ACLR has been shown with level 1 evidence to reduce anterolateral rotational laxity and clinically relevant reduction in graft rupture [10]. This is the most frequently used LET technique and both biomechanical and clinical studies have supported its efficacy [50].

Compared to the original one, this technique typically requires a shorter, narrower ITB strip that remains attached distally at Gerdy’s tubercle but is not routinely returned to the tibia. The graft is passed deep to the LCL (often using the LCL as a pulley), then fixed to the femur at a point proximal and posterior to the LCL’s femoral attachment (commonly near the lateral epicondylar region). Fixation may be achieved with staples, interference screws, or suture anchors, and the graft is secured under low tension with the knee positioned between 30 and 60 degrees to minimize an increased constraint of physiologic motion, while preserving a meaningful restraint to pivot-shift–type rotatory laxity.

#### Original Lemaire Procedure

The classic Lemaire tenodesis [18] uses a long, distally based strip of iliotibial band (ITB) left attached at Gerdy’s tubercle to create a lateral restraint against internal tibial rotation. After harvesting the strip, it is routed deep to the lateral collateral ligament (LCL), then redirected through femoral and tibial bony pathways so the graft effectively “wraps” around the lateral side of the knee before final fixation. The construct is tensioned and fixed with the knee in limited flexion and neutral rotation to avoid excessive lateral compartment constraint, aiming to provide an extra-articular checkrein that complements intra-articular ACL reconstruction.

#### Ellison Procedure

The Ellison technique [26, 51] differs from most ITB-based tenodeses by performing a distal transfer. A small bone fragment is elevated from Gerdy’s tubercle with the attached ITB strip, allowing controlled relocation of the distal attachment. The lateral capsule may be addressed with plication and the transferred graft is then routed deep to the LCL and secured anteriorly in a bony trough. Conceptually, the procedure attempts to restore anterolateral rotational control by re-tensioning the ITB rather than relying solely on proximal femoral fixation.

#### MacIntosh Lateral Substitution (Over-the-Top)

MacIntosh’s lateral substitution over-the-top reconstruction [51] uses a large, distally based ITB graft and combines intra-articular and extra-articular components in a continuous pathway. After the graft is passed deep to the LCL, it is shuttled through a subperiosteal tunnel and then directed around the lateral femoral condyle in an “over-the-top” manner, entering the joint and ultimately returning through a tibial tunnel back toward the anterolateral tibia/ITB insertion area. Multiple fixation points are used along the route to stabilize the construct, with the goal of addressing both anteroposterior and rotatory instability patterns.

#### “Over-the-Top” Hamstring LET

The ‘Marcacci approach’ [26, 52] achieves combined ACL reconstruction and lateral tenodesis using both semitendinosus and gracilis tendons, left attached distally at the pes insertion. The graft is passed through a tibial tunnel intra-articularly, redirected around the lateral femoral condyle in an over-the-top fashion, and brought out laterally adjacent to the ITB. Proximal fixation is commonly performed near the lateral femoral cortex with the knee flexed, and the remaining graft is then routed distally to create a lateral tenodesis component, often secured near or just below Gerdy’s tubercle. The concept is useful in young, highly active, or selected skeletally immature patients when appropriately adapted but there are literature reports supporting its usage in adults as well [52].

#### Arnold–Coker Modification of the MacIntosh Technique

This all-soft-tissue modification [8, 26, 51] harvests a narrower ITB strip distally attached at Gerdy’s tubercle, passes it deep to the LCL, and then reflects it back to be sutured onto itself under tension with the knee flexed at 90 degrees. A key technical feature is setting the tibia in external rotation during fixation to maximize tightening of the fascial strip and enhance rotatory control. It is often described as a simplification of older MacIntosh-style reconstructions that used more extensive osseo-periosteal tunneling and looping.

#### Micheli – Kocher Technique (Pediatric ITB-based, Physeal-Sparing Concept)

The Micheli-Kocher method [8, 26, 51] is an ITB-based reconstruction designed for skeletally immature patients, aiming to avoid bone tunnels for both the ACL and the lateral augmentation. A long ITB graft remains attached distally at Gerdy’s tubercle, is tubularized, and then passed through an over-the-top femoral route and a tibial “over-the-front/under the intermeniscal ligament” pathway to recreate intra- and extra-articular restraint. Fixation is performed with sutures to soft tissues or the periosteum at knee positions chosen to balance stability with avoidance of growth-plate injury. The technique is aimed at providing a tunnel-free strategy for combined stability restoration in carefully selected pediatric cases.

#### Anterolateral ligament reconstruction

Anterolateral ligament reconstruction is typically performed as an anatomic, graft-based reconstruction of the ALL to restore anterolateral restraint. Various technical approaches have been described using a multitude of grafts, with the most common being the gracilis autograft [8]. Key landmarks include the fibular head, Gerdy’s tubercle, and the lateral femoral epicondyle. A femoral incision is created slightly proximal and posterior to the lateral epicondyle, and a tibial incision is placed ~ 1 cm distal to the joint line between Gerdy’s tubercle and the fibular head. A doubled gracilis autograft is frequently used, reflecting its favorable mechanical behavior for replicating ALL functions [4, 9, 19]. Fixation can be achieved using screws, staples, sutures, or anchors, including knotless soft anchors, which may help mitigate tunnel convergence by using smaller tunnels. Importantly, graft tensioning and fixation should be performed with the knee in full extension and the tibia in neutral rotation [14]. Femoral fixation is placed proximal and posterior to the lateral femoral epicondyle to reproduce anatomic anisometry (tight in extension, slack in flexion) and reduce over constraint risk. Depending on the overall reconstruction strategy and tunnel configuration, ALLR can be performed with an independent tunnel set separate from the ACL reconstruction, or with a continuous one, to reduce tunnel conflict and improve reproducibility [8, 53].

#### Is there a superior technique?

At present, the literature does not support a single “superior” LET technique that consistently outperforms all others across indications. This is also reflected in the statement of the International Consensus, which emphasizes that multiple LET options remain acceptable and that the principles of the procedure likely matter more than the name of the technique. In practice, most modern LETs are variations of ITB-based constructs, and the available biomechanical work suggests that a graft route that preserves a favorable lever arm against tibial internal rotation—typically by routing the strip deep to the LCL—can better approximate native kinematics and reduce excessive graft tightening or slackening across flexion. However, clinical head-to-head comparisons between different LET constructs are scarce, and outcome differences (PROMs, return to sport, failure etc.) appear to be driven more by patient selection, correct femoral fixation point, neutral-rotation fixation, and avoiding over-tensioning than by the specific construct chosen [53].

Where technique selection does become more “personalized” is by age and skeletal maturity. In skeletally immature patients, the “best” LET is often the one that can be performed safely without physeal injury, favoring physeal-sparing adaptations and ITB-based options designed for pediatric anatomy. In adolescents nearing maturity, surgeons may use modified adult-style ITB tenodeses but still tailor the fixation method, location and tensioning to minimize an increased constraint of physiologic motion and hardware irritation. In adults, the choice often comes down to reproducibility and surgeon familiarity. Taken together, the current state of evidence supports an approach that prioritizes standardized surgical principles and age-appropriate modifications rather than claiming one universally superior LET technique.

### Author’s Approach

**Fig. 1:**
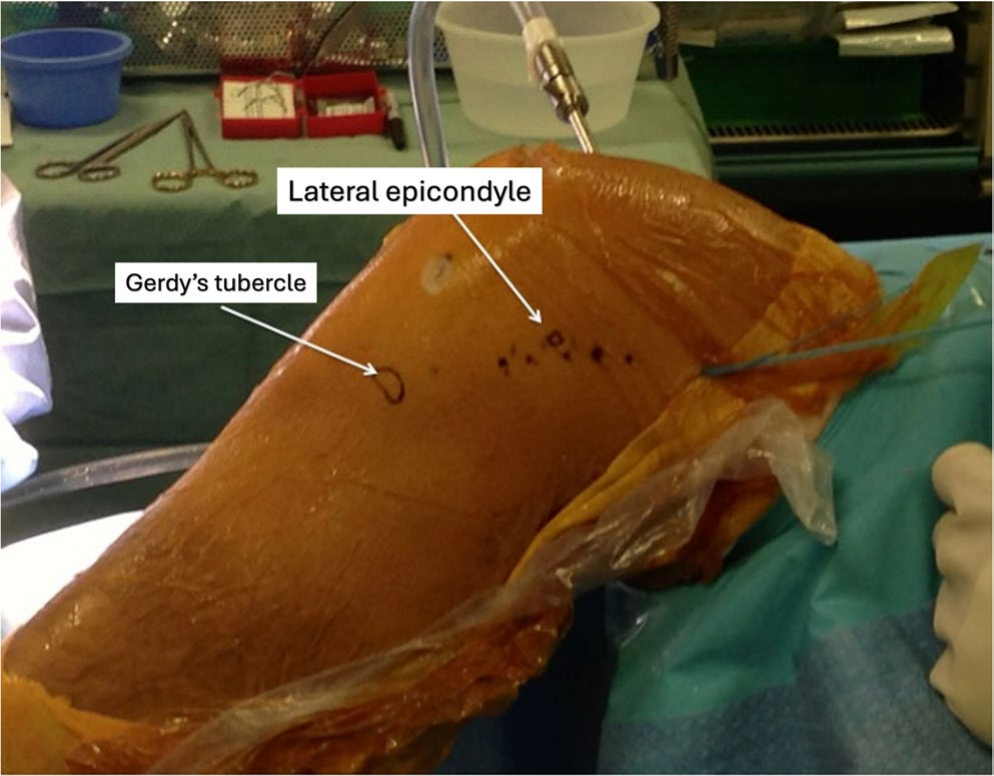
After identifying the lateral epicondyle and Gerdy’s tubercle, a longitudinal lateral incision of roughly 6 cm is made just posterior to the lateral femoral epicondyle to expose the ITB

In our practice, the preferred method is a modified Lemaire lateral extra-articular tenodesis performed after completion of the intra-articular ACL reconstruction. With the patient supine, no tourniquet applied and standard arthroscopy set-up with both lateral and flexion footposts. The LET is undertaken once the ACL graft has been passed, tensioned, and fixed. The knee is flexed to approximately 90°, and a longitudinal lateral incision (roughly 6 cm) is made just posterior to the lateral femoral epicondyle to expose the ITB (Fig. 1).

**Fig. 2:**
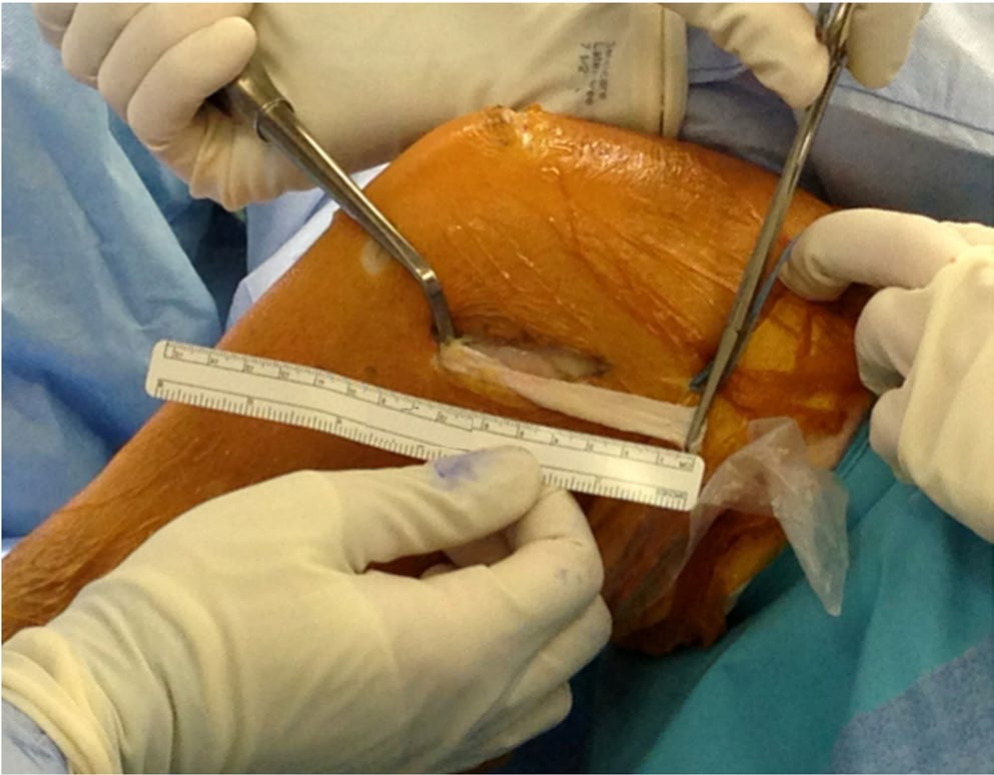
An ITB strip approximately 8 cm long and 1 cm wide is developed and freed from deep attachments while preserving its distal insertion at Gerdy’s tubercle

A distally based ITB strip is then harvested to create the tenodesis graft (Fig. 2). After identifying the posterior margin of the ITB, an ITB strip approximately 8 cm long and 1 cm wide is developed, released proximally, and freed from deep attachments while preserving its distal insertion at Gerdy’s tubercle. The proximal end is reinforced with a whipstitch. Care is taken to avoid harvesting too posteriorly to protect the posterior ITB fibers.

**Fig. 3:**
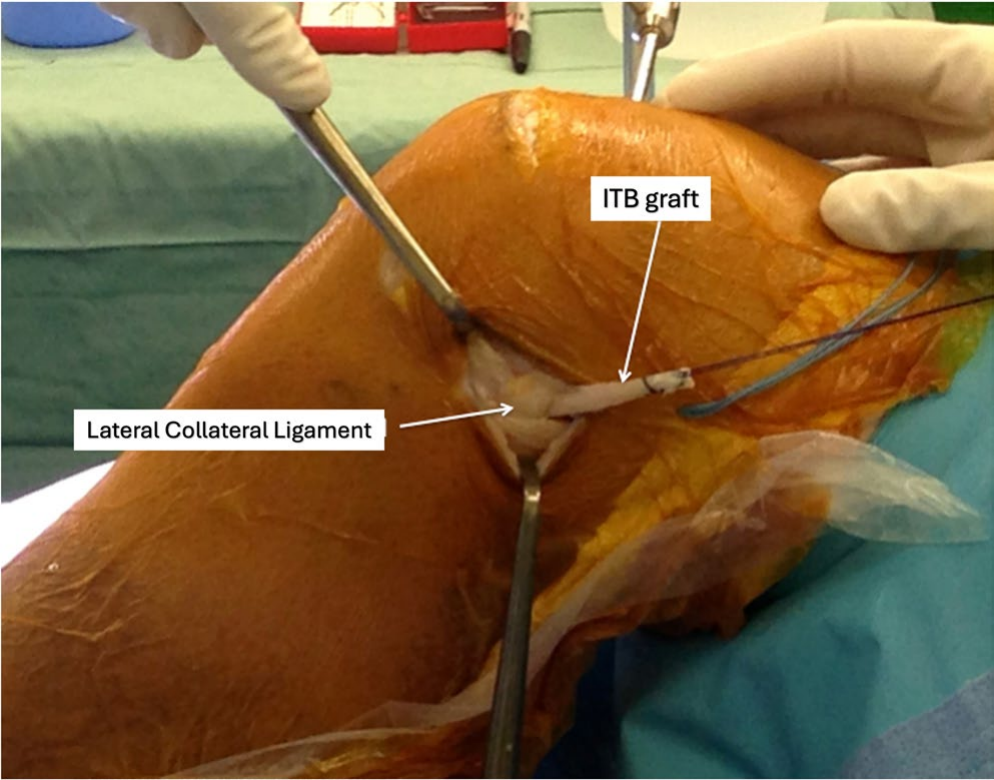
The proximal end of the ITB graft is reinforced with a whipstitch and is then passed from distal to proximal beneath the LCL

The lateral collateral ligament is identified by palpation (often facilitated by a figure-of-four position), and a soft-tissue tunnel is created deep to the LCL using small incisions anterior and posterior to its proximal aspect. Dissection is kept extra-capsular to avoid injury to the popliteus tendon. The ITB graft is then passed from distal to proximal beneath the LCL using a clamp (Fig. 3).

**Fig. 4:**
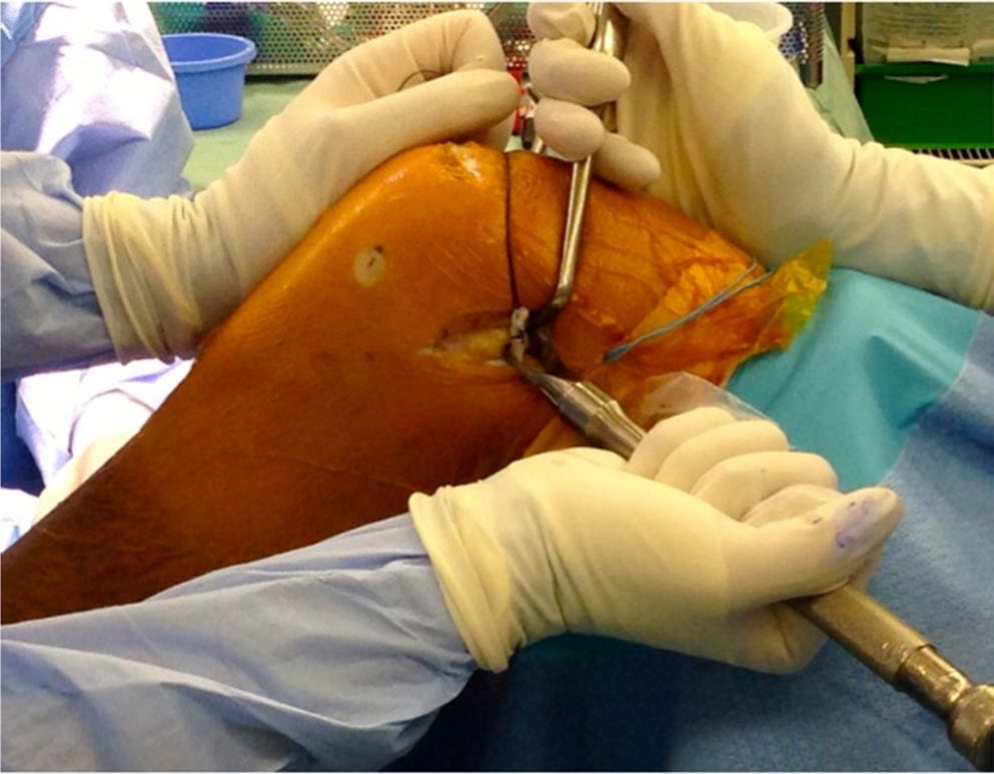
The graft is seated with minimal tension and secured to the femur with a staple while the knee is positioned at 60° of flexion with the foot in neutral rotation

**Fig. 5:**
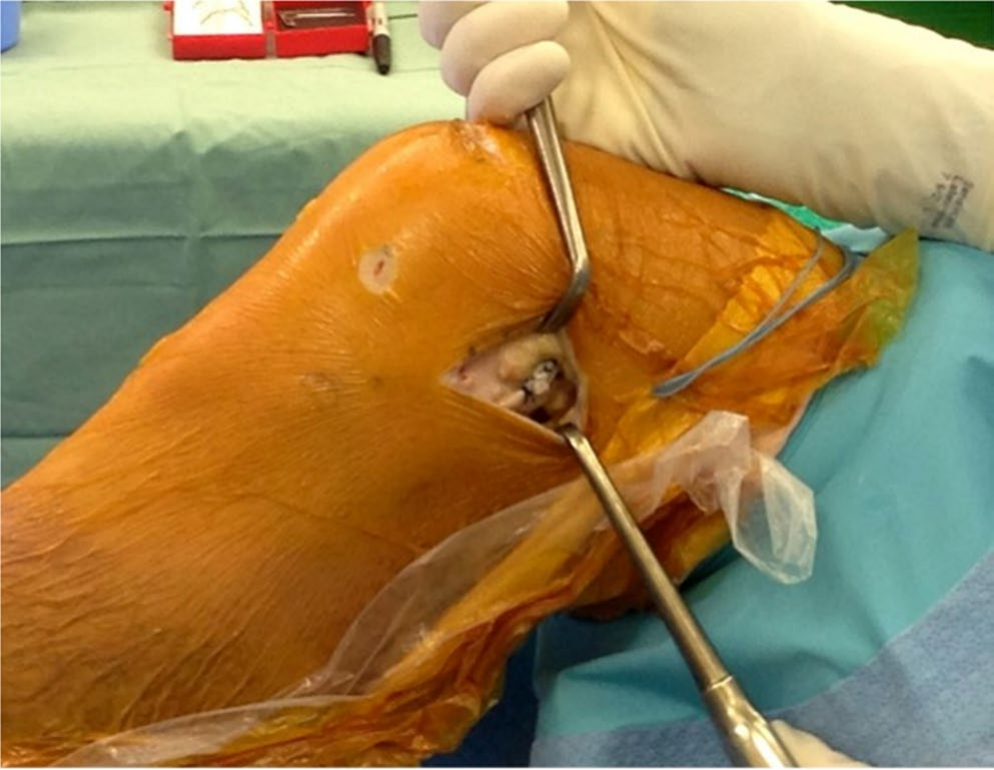
Any redundant graft can be folded back and sutured onto itself to help avoiding irritation during range of motion

Femoral fixation is performed at a reproducible “isometric” region aimed as posterior and proximal to the femoral origin of the LCL and just anterior to the distal Kaplan fiber attachment on the supracondylar flare. The periosteum is cleared with a Cobb retractor, and the knee is positioned at ~ 60° of flexion with the foot in neutral rotation. The graft is seated with minimal tension and secured to the femur with a staple or knotless suture anchor (Fig. 4). Any redundant graft can be folded back and sutured onto itself. The ITB defect is then re-approximated proximally, while the distal ITB split is typically left unclosed to reduce the risk of lateral patellofemoral over-constraint (Fig. 5).

Postoperatively, weightbearing and range of motion are generally permitted as tolerated within the standard ACL rehabilitation pathway, with modifications only when dictated by concomitant meniscal or cartilage procedures.

### Future Directions

Future work on LET is likely to move from “does it help?” toward “who benefits most, with which construct, and at what cost.” Key directions might include prospective, technique-to-technique trials using standardized tensioning and positioning to isolate true construct effects; [2] precision indications built from multivariable risk models (age, sport exposure, laxity phenotype, posterior tibial slope, hyperextension, meniscal status, revision setting) rather than broad categories; and [3] better objective rotatory-laxity quantification (inertial sensors, navigation/robotic measurements, standardized pivot-shift grading) linked to patient-reported outcomes and re-injury. Additional priorities are long-term surveillance for lateral-compartment osteoarthritis and agreement on reporting standards (graft dimensions, fixation site, tension, knee position, complications) so results can be compared across studies and incorporated into future consensus updates.

## Conclusion

When applied selectively in high-risk patients, adding a lateral extra-articular tenodesis to ACL reconstruction can improve rotational control and reduce re-injury, but current evidence does not support a single universally superior LET construct. Optimal results depend on appropriate indications, careful technique, and tailoring the approach to patient factors such as age, sport demands, laxity profile, and skeletal maturity.

## Key References

- Zabrzyńskiet al. Technical Variations in Lateral Extra-Articular Tenodesis for Anterior Cruciate Ligament Reconstruction: A Systematic Review. J Clin Med. 2025 Sep 16;14(18):6510.
  - ◦ This systematic review clearly maps the current heterogeneity of LET techniques (graft choice, fixation points, fixation methods, knee angle and tensioning) and highlights why standardized reporting and technique clarity are necessary when interpreting outcomes across studies.
- Sonnery-Cottet et al. Indications for Lateral Extra-articular Procedures in the Anterior Cruciate Ligament-Reconstructed Knee: Part I of an International Consensus Statement. Arthroscopy. 2025 Sep;41(9):3303-3312.
- Sonnery-Cottet et al. Surgical Treatment and Complications of Lateral Extra-articular Procedures in the Anterior Cruciate Ligament-Reconstructed Knee: Part II of an International Consensus Statement. Arthroscopy. 2025 Sep;41(9):3313-3321.
  - ◦ This is a contemporary, international Delphi consensus that provides clear, practice-oriented indications for adding a lateral extra-articular procedure (LEAP/LET/ALLR) to ACLR. Part one focuses also on indications, such as high-grade pivot shift, young active patients, revision, hyperextension, slope thresholds, making it ideal for justifying patient selection. Part II supports the technical and safety framing by summarizing core technical principles of lateral extra-articular procedures and states that no single technique is clinically superior, with low complication rates, no demonstrated increased lateral OA risk, and no need to alter rehab protocols.
- Gunturu et al. The Anterolateral Ligament of the Knee: Anatomical, Biomechanical, and Clinical Perspectives with Implications for Injury Management. Orthopedics. 2026 Jan-Feb;49(1): e101-e107.
  - ◦ This recent narrative review provides a concise, up-to-date synthesis of ALL anatomy, biomechanical function, and clinical relevance, helping readers understand the conceptual rationale for anterolateral augmentation in ACL-deficient knees and why the topic remains actively debated.
- D'Ambrosi et al. Combining an Anterolateral Complex Procedure With Anterior Cruciate Ligament Reconstruction Reduces Graft Reinjury Without Increasing the Rate of Complications: A Systematic Review and Meta-analysis of Randomized Controlled Trials. Am J Sports Med. 2025 Aug;53(10):2462-2470.
  - ◦ This meta-analysis of RCTs provides high-level clinical evidence that adding an anterolateral procedure can reduce graft failure and reinjury rates while not increasing complications, making it a strong, outcomes-focused reference to support the clinical relevance and safety of combined procedures.

## Data Availability

No datasets were generated or analysed during the current study.
